# Effect of Alloying Elements in Steels on the Interfacial Structure and Mechanical Properties of Mg to Steel by Laser-GTAW Hybrid Direct Lap Welding

**DOI:** 10.3390/ma17071624

**Published:** 2024-04-02

**Authors:** Xin Liu, Qiang Lang, Jifeng Wang, Gang Song, Liming Liu

**Affiliations:** 1 School of Materials Science and Engineering, Dalian University of Technology, Dalian 116000, China; bjt450137664@163.com (X.L.); lqiang_dlut@163.com (Q.L.); liulm@dlut.edu.cn (L.L.); 2 Shanghai Institute of Special Equipment Inspection and Technical Research, Shanghai 200062, China; dishengwang@126.com

**Keywords:** Mg/steel welding, laser-GTAW hybrid direct lap welding, interfacial structure, mechanical properties

## Abstract

Mg alloy AZ31B was directly bonded to SK7 with a low alloy content, DP980 with a high Mn content, 316L with a high Cr and high Ni content by laser-gas tungsten arc welding (GTAW) and hybrid direct lap welding. The results showed that the tensile loads of AZ31B/SK7 and AZ31B/DP980 joints were 283 N/mm and 285 N/mm respectively, while the tensile load of AZ31B/316L joint was only 115 N/mm. The fracture and interface microstructures were observed using scanning electron microscopy (SEM), electron probe microanalysis (EPMA), and identified through X-ray diffractometry (XRD). For AZ31B/SK7 and AZ31B/DP980, the interface of the front reaction area and the keyhole reaction area was mainly composed of an Fe-Al phase and an Al-Mn phase. However, for AZ31B/316L, the interface of the keyhole reaction area was mainly composed of an Fe-Al phase and an Al-Mn phase, but a multi-layer composite structure consisting of the Mg_17_Al_12_ compound layer and eutectic layer was formed in the front reaction area, which led to a deterioration in the joint property. The influencing mechanism of Mn, Cr and Ni elements in steel on the properties and interface structure of the laser-GTAW lap joint between the Mg alloy and the steel was systematically analyzed.

## 1. Introduction

The inevitable trend in automotive manufacturing’s future lies in the integration of lightweight structures into automobiles [1,2]. Within the realm of automobile production, optimizing structural design and reducing body weight can significantly mitigate fuel consumption and carbon emissions associated with vehicles. Mg alloy, being the lightest engineering material available, possesses inherent advantages such as exceptional electrical and thermal conductivity, high specific strength, remarkable damping capabilities, shock absorption properties, as well as facile recyclability [3,4]. However, the predominant use of steel as the key structural material in automobile manufacturing persists, thanks to its inherent advantages such as exceptional strength, admirable heat resistance, and ease in fabricating intricate structures [5,6]. Therefore, the integration of these two materials in a composite structure offers a viable solution to reducing body weight without compromising structural integrity, necessitating the development of advanced welding technologies for Mg and steel as well as the promotion of design and application of heterogeneous Mg/steel components.

Due to the substantial disparities in physical and chemical properties between Mg and Fe, their lack of reactivity hinders the formation of the solid solution and compounds, posing challenges for its bonding [7,8,9,10]. In previous studies, the incorporation of alloying elements into Mg and Fe was commonly achieved through the addition of coatings, interlayers, or filling wires capable of forming intermetallic compounds (IMCs) or solid solutions. Xu et al. [11] achieved lap welding of AZ31 and Q235 by incorporating a Zn–Al coating during friction stir spot welding (FSSW), resulting in the formation of an Al_5_Fe_2_ IMC layer at the lap interface. The joint exhibited a tensile load capacity of 143.3 N/mm. Chen et al. [12] successfully achieved lap welding between a ZEK100 Mg alloy and DP600 steel using refill friction stir spot welding (RFSSW). It was observed that the immersion of a zinc solution containing 0.15–0.19% Al on the DP600 plate resulted in the formation of FeAl_2_ particles at the interface. These particles serve as an intermediate layer between the Mg alloy and steel grains, contributing to an average maximum bonding strength of 188 N/mm in the joint. Tan et al. [13] successfully achieved lap welding of an AZ31B–H24 Mg alloy and stainless steel through the utilization of a dual-beam laser welding-brazing technique, employing a filler material composed of Mg-Al-Zn wire with identical composition to the Mg base metal. A continuous thin reaction layer, identified as FeAl IMC, was formed along the interface between Mg and Fe, resulting in a maximum joint strength of 274.5 N/mm. Liu et al. [14] successfully achieved lap welding between AZ31B and DP600 using resistance spot welding (RSW). This achievement can be attributed to the formation of nanoscale Fe_3_Al IMC at the interface, of which Al is from an AZ31B base material, resulting in a coherent or semi-coherent interface on both sides of the weld. The lap joint exhibited an impressive welding strength of 228 N/mm. Li et al. [15] investigated the laser welding-brazing process of AZ31B/stainless steel lap welding filled with AZ31 wire, and a joint strength reached a maximum of 270 N/mm. It was observed that the Mg/steel interface exhibited an enrichment of aluminum elements, leading to the formation of a transition layer approximately 1.5 μm thick, which significantly contributed to enhancing the joint properties. It was widely believed that the formation of Al-based compounds or solid solutions played a crucial role in achieving high-performance Mg/steel joints [16]. In conclusion, it was imperative to investigate the influence of alloying elements on interfacial reactions. However, previous literature has primarily focused on elucidating the role of alloying elements in filling materials during the welding process, aiming to enhance the spreading wettability of Mg alloys on steel or promote metallurgical bonding between the Mg alloy and steel by reacting with Mg or Fe. Nevertheless, limited comparative research has been conducted regarding the effects of alloying elements in steel on the properties of Mg/steel joints.

Low-power pulsed laser-gas tungsten arc welding (GTAW) hybrid welding technology, based on previous experiments, has been identified as one of the effective approaches for achieving high-strength bonding between Mg alloy and steel. This innovative technique enables precise spatial energy distribution between the pulsed laser and the arc heat source. Moreover, the utilization of a low-current arc minimizes the evaporation and burning loss of the Mg alloy, while the pulse laser partially melts the steel to create a keyhole with a specific depth that nails the weld. The lap joints of Mg/steel obtained by laser-GTAW hybrid welding technology exhibit distinctive interface structural characteristics, with more intricate interface reactions, so comprehensive research is urgently required.

Different from the traditional welding methods, which need to add interlayer, coating and wire feeding, a new laser arc direct welding method of magnesium steel is proposed in this paper. The space position of the laser and arc heat source, that is, the malposition, is adjusted to achieve high-performance welding of magnesium steel. With this new welding method, the influences of the main alloying elements in different steels (SK7 with a low alloy content, DP980 with a high Mn content, 316L with a high Cr and high Ni content) on the microstructure and mechanical properties of magnesium steel joints were studied.

## 2. Materials and Methods

### 2.1. Materials

In the experiment, base metals including a 1.5 mm-thick Mg alloy AZ31B, a 1.2 mm-thick carbon tool steel (SK7), a 1.6 mm-thick high-strength dual phase steel (DP980), and a 1.0 mm-thick austenitic stainless steel (316L) were utilized. The chemical composition of the substrates is presented in Table 1. The types and contents of alloying elements of the three kinds of steel were different, among which the alloying elements of SK7 were less, the Mn content of DP980 was higher, and the Cr and Ni content of 316L was higher. The plates were cut into samples measuring 60 mm × 100 mm for welding. Before welding, the surfaces of the samples were treated with alcohol (Tianjin Fuyu Fine Chenmical Co., Ltd., Tianjin, China) and acetone (Tianjin Fuyu Fine Chenmical Co., Ltd., Tianjin, China) to remove grease and rust, followed by polishing to eliminate any surface oxide film present.

### 2.2. Experimental Method

**Figure 1:**
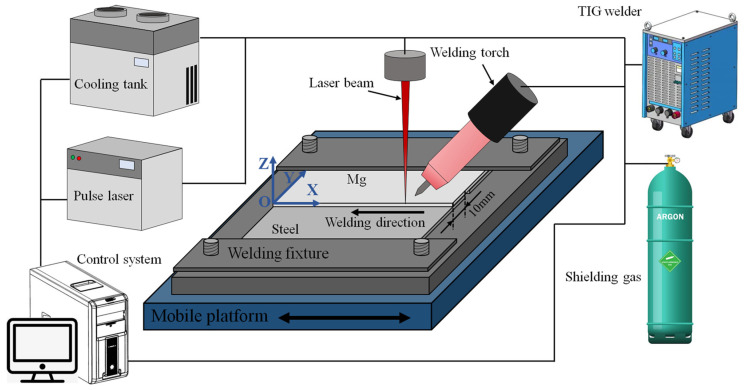
Schematic illustration of lap joining for Mg/steel by laser-GTAW.

The welding equipment was a hybrid system consisting of a Nd: YAG (Neodymium-doped Yttrium Aluminium Garnet; Nd: Y3Al5O12, Guangzhou Ruitong Laser Technology Co., Ltd., Guangzhou, China) pulsed solid-state laser with a maximum power of 1 kW and a GTAW machine (OTC AVP-500, OTC Electromechanical Qingdao Co., Ltd., Qingdao, China) with a peak current of 500 A. The laser had an infrared wavelength of 1064 nm, a focal distance of 120 mm, and a minimum spot diameter of 0.6 mm, which was similar to other lasers used in industrial applications. The tungsten electrode of the arc was WC20 with a diameter of 2.4 mm. The sample to be welded was fixed on a movable platform, and the experimental setup is shown in Figure 1. The welding process utilized a 10 mm wide lap structure, with the upper layer consisting of Mg alloy and the lower layer being made of steel. The laser was directed perpendicularly onto the upper surface of the Mg alloy, while the TIG welding torch was angled at 45° to the laser beam in the X-O-Z plane. This paper innovatively adopted non-coaxial welding between the laser and arc heat source; that is, the distance between the laser beam and tungsten tip in the O-X direction was 1.5 mm, and the distance between the laser beam and tungsten tip in the O-Y direction was 1.0 mm. The optimized welding parameters are shown in Table 2.

### 2.3. Characterization Method

After welding, the tensile specimens were prepared in accordance with the national standard (GB/T 228.1-2010 [17]) along the vertical welding direction. Tensile tests were conducted at room temperature using an electronic universal testing machine (Instron 5982, INSTRON CORPORATION, Boston, MA, USA) at a constant speed of 1 mm/min. Three identical samples were tested for each parameter, and the average value was considered as the test result. The macroscopic morphology, cross-section and fracture path of the joint were studied using a scanner (EPSON perfect V39, Seiko Epson Corporation, Suwa, Nagano-ken, Japan) and an optical microscope (OM, Leica MEF4, Shanghai Tuming Optical Instrument Co., Ltd., Shanghai, China). The microstructure morphology, elemental analysis, and phase analysis of the fracture surface were examined using field emission scanning electron microscopy (SEM, SUPRA55, Zeiss Optical Instruments Ltd., Oberkochen, Baden-Württemberg, Germany) at 10 kV and X-ray diffractometry (XRD, D8 Advance, Brooke (Beijing) Technology Co., Ltd., Beijing, China). Additionally, the microstructure morphology and energy spectrum of the weld cross-section were observed and analyzed using a field emission electron probe micro-analyzer (EPMA, JXA-8530F PLUS, JAPAN Electron OPTICS LABORATORY Co., Ltd., Akishima-shi, Tokyo, Janpan) equipped with an energy dispersive spectrometer (EDS) at 10 kV.

## 3. Results and Discussion

### 3.1. Macroscopic Morphology

**Figure 2:**
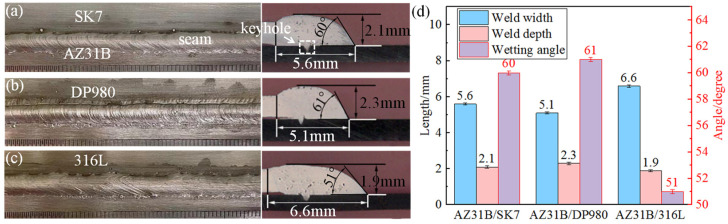
Three-dimensional macroscopic morphology and cross-sectional morphology of different joints: (**a**) AZ31B/SK7; (**b**) AZ31B/DP980; (**c**) AZ31B/316L; (**d**) weld width, weld depth, and wetting angle of the three joints.

The macroscopic surface morphology and cross-sectional morphology of AZ31B/steels (SK7, DP980, and 316L) joints obtained under identical experimental parameters are illustrated in Figure 2. According to the surface morphology of welded joints, Mg/steel joints with continuous forming and no defects were obtained. According to the cross-sectional morphology, the keyhole was formed under the action of the laser. The weld width, weld depth, and wetting angle of the three joints were different. AZ31B/316L had the smallest wetting angle (51°), followed by AZ31B/SK7 (60°), and AZ31B/DP980 had the largest wetting angle (61°). Correspondingly, the weld width decreased successively and the weld depth increased successively, as shown in Figure 2d. The smaller the wetting angle and the larger the weld width were, the larger was the spreading area of the Mg alloy on the steel, which indicated that the spreading wettability of the magnesium alloy on 316L was better, which may be attributed to the presence of Cr and Ni. Studies have shown that Cr [18] promoted the formation of a smooth passivation film on the surface of the steel, and a smooth surface on the steel meant that the surface tension of the solid steel was greater. According to Young’s equation [19,20]:

(1) $$\gamma_{sv}-\gamma_{sl}=\gamma_{lv}\cos\theta$$

In the equilibrium state, a smaller wetting angle tended to occur with a greater surface tension γ_sv_ at the solid-vapor interface. Additionally, it has been demonstrated that the addition of Ni is beneficial to the spreading wetting of the Mg alloy [21].

### 3.2. Tensile Test

**Figure 3:**
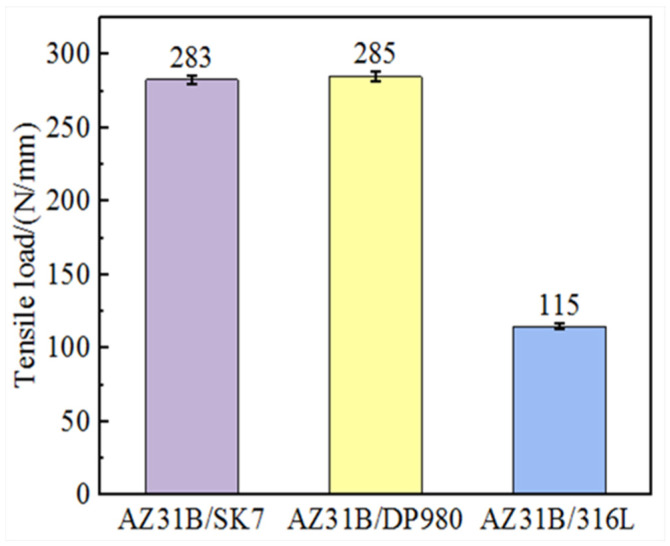
Maximum average tensile load of the three joints.

The tensile load of AZ31B/steel (SK7, DP980, and 316L) joints was tested under identical process parameters to compare the influence of different elements on the mechanical properties of the joint, and the results are presented in Figure 3. It was evident that the tensile load of AZ31B/SK7 was slightly lower at 283 N/mm compared to that of AZ31B/DP980, which achieved a value of 285 N/mm. Conversely, the AZ31B/316L exhibited the smallest tensile load of only 115 N/mm, but it demonstrated a larger interface bonding area and a smaller wetting angle in Figure 2c. As Mg and Fe did not interact with each other, the wettability of the melt would be a key factor that determined the bonding state [22] between the FZ and the steel. The results showed that the joint with the best wettability had the worst mechanical properties, so the joint properties of the Mg/steel were not solely determined by the spreading and wetting behavior. Previous studies have highlighted that the interface strength resulted from a synergistic interplay of various factors, including a reduced wetting angle, obtaining a continuous interface layer at the micro and nano scales, and a larger bonding area at the interface, all contributing to enhanced joint strength [23]. Therefore, the differences in mechanical properties of the three joints may be attributed to the differences in the interface structure and interface microstructure, which were verified by subsequent experiments.

**Figure 4:**
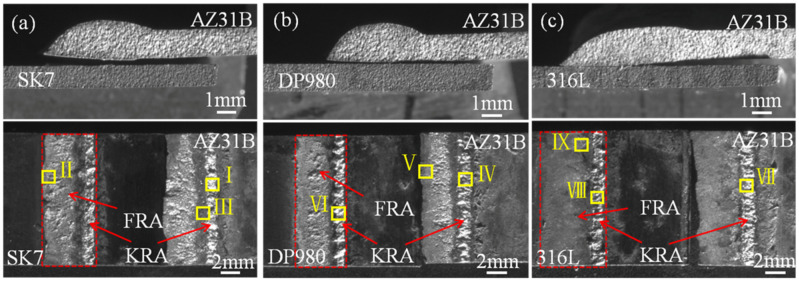
Fracture paths and fracture surfaces: (**a**) AZ31B/SK7; (**b**) AZ31B/DP980; (**c**) AZ31B/316L.

The fracture modes of these three joints were all along the bonding interface between the Mg alloy and steel. And the fracture path and fracture surface are shown in Figure 4. It is noteworthy that the fracture surfaces exhibited distinct metallurgical regions (red boxes), which aligned with previous findings and were closely associated with the thermal gradient distribution within the joint [23]. According to the thermal gradient distribution, the joint could be divided into two areas, namely the weld front reaction area (FRA) and the keyhole reaction area (KRA), respectively.

**Figure 5:**
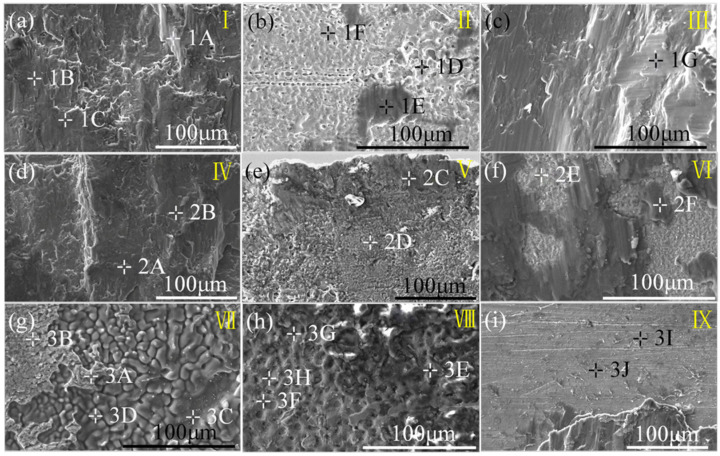
Fracture surface microstructure: (**a**–**c**) AZ31B/SK7; (**d**–**f**) AZ31B/DP980; (**g**–**i**) AZ31B/316L.

The locations in different areas marked by the yellow boxes in Figure 4 underwent microscopic magnification as presented in Figure 5 and quantitative point analysis results which are shown in Table 3, Table 4 and Table 5. I–IX in Figure 4 correspond to I–IX in Figure 5. Based on binary phase diagrams of Al-Mg, Al-Fe, and Al-Mn [24,25,26], Fe_3_Al IMCs were both formed at the KRA of AZ31B/SK7 and AZ31B/DP980, while Al_11_Mn_4_ IMCs were observed at the KRA of the AZ31B/DP980 with a higher Mn content. These findings have been previously reported [27,28] and further validated through micro XRD analysis conducted during this study. It was suggested that fracture along Mg/Fe may be related to the thickness of the interface layer. Different from the fracture morphology of the other two joints (Figure 5b,e), the fracture surface of the FRA of AZ31B/316L (Figure 5e) was smoother. Based on the quantitative point analysis results, it can be inferred that the brittle eutectic structure consisting of Mg_17_Al_12_ + (Mg) and Mg_17_Al_12_ IMCs (Table 5) may account for the low strength and brittle fracture behavior.

### 3.3. Microstructure

**Figure 6:**
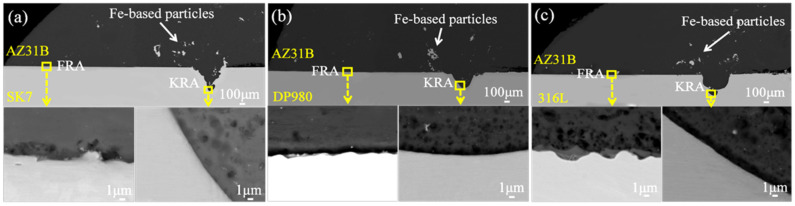
The interface microstructure of the joints: (**a**) AZ31B/SK7; (**b**) AZ31B/DP980; (**c**) AZ31B/316L.

The microstructure of these different types of joints and the Mg/Fe interfaces at both FRA and KRA are depicted in Figure 6. The weld exhibited massive nano-scale Fe-based particles in the Mg/Fe interface of the three joints. The dispersed Fe-based particles can be attributed to the overflow of molten metal from the keyhole during pulsed laser welding, which, due to insufficient time for complete expulsion during the cooling and solidification, resulted in their retention within the weld. However, a multi-layer structure was observed at the FRA of the AZ31B/316L joint and no obvious interface layer was observed in the FRA and KRA of the AZ31B/SK7 and AZ31B/DP980 joints.

**Figure 7:**
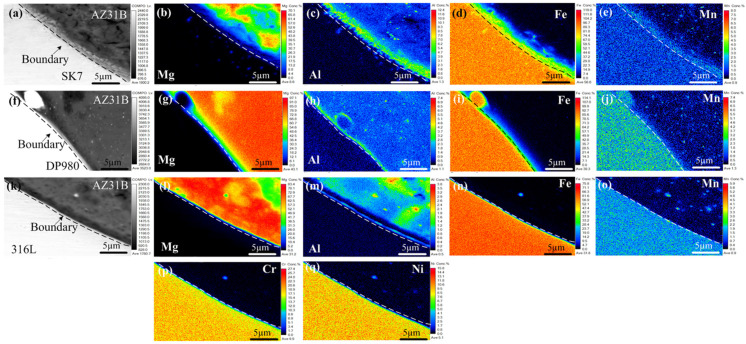
Mapping analysis of the Mg/Fe interface at the KRA: (**a**–**e**) AZ31B/SK7; (**f**–**j**) AZ31B/DP980; (**k**–**q**) AZ31B/316L.

The KRA of the three joints in Figure 6 was subjected to EPMA mapping analysis to further investigate the distribution of elements and chemical composition within the interface layer, as depicted in Figure 7. Notably, a nanometer-scale thickness interface layer with Al element segregation (Figure 7c,h,m) was observed at the Mg/Fe interface for all three joints examined. This was similar to the interface layer formation observed during laser welding-brazing [29], where Fe-Al IMCs formed due to segregation and reaction between Al and Fe elements. The Mg/Fe interface of the three joint keyholes exhibited an uneven granular distribution of Mn, and Al had a similar distribution with Mn. The results showed that new compounds or solid solutions at the nanoscale may be formed. All of this meant that metallurgical bonding was achieved between the Mg and steel, which had a positive effect on the enhancement of the mechanical properties of the joint. As shown in Figure 7p,q, no noticeable enrichment of Cr and Ni was observed at the keyhole interface in the AZ31B/316L, suggesting that these two alloying elements did not directly participate in the reaction at the interface.

**Figure 8:**
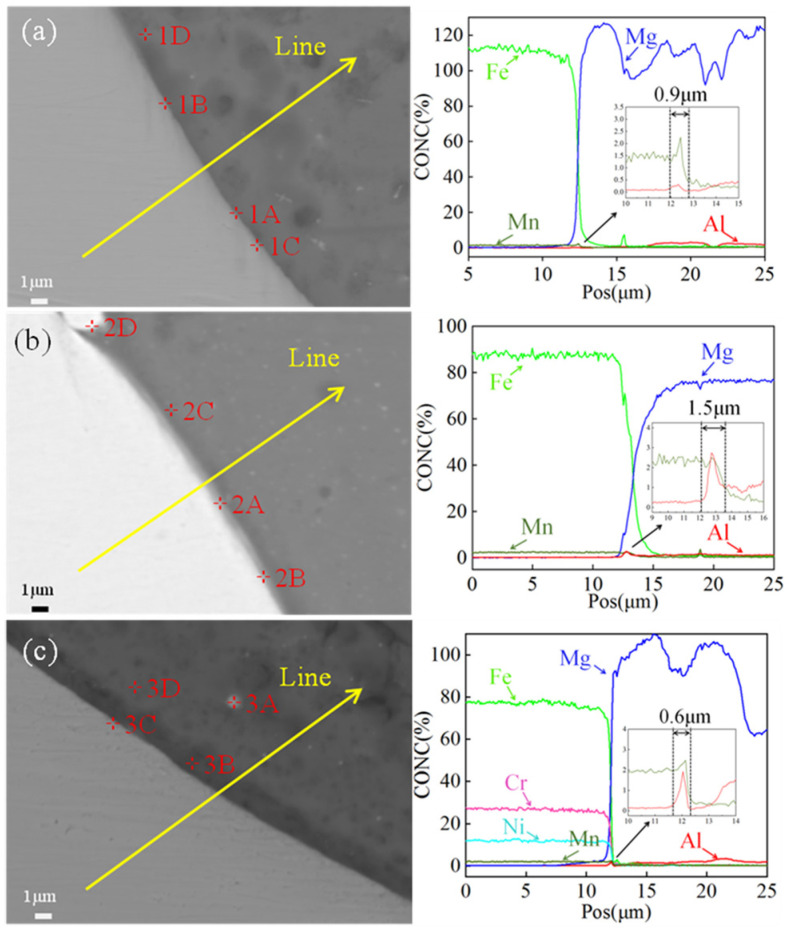
Line analysis of the Mg/Fe interface at the KRA: (**a**) AZ31B/SK7; (**b**) AZ31B/DP980; (**c**) AZ31B/316L.

The line analysis results of the Mg/Fe interfaces at the keyhole regions of three types of joints are illustrated in Figure 8. The Al contents exhibited an initial increase followed by a decrease, attributed to segregation at the interface. Similarly, the Mn contents displayed a distribution trend consistent with that of the Al at the interface, confirming the formation of an Al–Mn phase in the keyhole region. Furthermore, the variations in Cr and Ni contents in the AZ31B/316L were found to be in agreement with those observed for Fe. The thickness of the interface layer can be determined by observing the variation trend of each constituent element. For the AZ31B/SK7 joint, the interface layer at the keyhole exhibited a thickness of 0.9 μm, while for the AZ31B/DP980 and AZ31B/316L joints, it measured 1.5 μm and only 0.6 μm, respectively. Within a certain range, an increased compound thickness at the interface was more advantageous for joint performance [30].

In order to gain further insights into its composition and structure, quantitative point analysis using EPMA was performed at various positions within the keyholes of three joints, and the corresponding results are presented in Table 6, Table 7 and Table 8. The elemental composition proportions indicated that there was an enrichment of Mn and Al at the interface in points 1A, 1C, and 1D (Table 6), which aligned with the findings from line analysis. According to the Fe–Al binary phase diagram [25] and Al-Mn binary phase diagram [26], it can be inferred that an (Fe) solid solution, Fe_3_Al, Al_11_Mn_4_, may form when Al from AZ31B combined with Fe or Mn from SK7. Additionally, Al-Mn compounds were preferentially formed over Al-Fe compounds according to the mixing enthalpy relationship [31], as evidenced by their respective mixing enthalpy values (Al-Ni < Al-Mn < Al-Fe < Al-Cr < Al-Mg < 0 J/mol). The compound Al_11_Mn_4_ exhibited two distinct crystal structures: high-temperature Al_11_Mn_4_ (HT-Al_11_Mn_4_), which belonged to the orthorhombic system, and low-temperature Al_11_Mn_4_ (LT-Al_11_Mn_4_), which belonged to the triclinic system. At a temperature of 1002 °C, Al reacted with Mn to form a high-temperature phase known as HT-Al_11_Mn_4_. Upon cooling, this phase underwent a transformation into a low-temperature phase called LT-Al_11_Mn_4_ at 910 °C. The reaction can be represented by the following formulas:

(2) $$L+\gamma_{1}\overset{1002{}^{\circ}C}{⇌}Al_{11}Mn_{4}(\mathrm{HT})$$

(3) $$Al_{11}Mn_{4}(\mathrm{HT})\overset{910{}^{\circ}C}{⇌}\mu +Al_{11}Mn_{4}(\mathrm{LT})$$

The LT-Al_11_Mn_4_ compound and the granular μ(Al_4_Mn) compound were formed, which was the source of the granular Al-Mn phase near the Mg/Fe interface on the Mg side. Consequently, a layered LT-Al_11_Mn_4_ was generated in the interface layer. The atomic radius of Fe is 126 pm, while that of Mn is 127 pm, and their atomic radii exhibit comparable magnitudes. Therefore, there was a high likelihood of Fe atoms substituting some Mn atoms, leading to the formation of an LT-Al_11_(Mn, Fe)_4_ IMCs-based solid solution. Point 1D was situated in the interface layer near the weld where it was probable that Fe_3_Al existed based on elemental composition and analysis of the binary phase diagram.

The points 2A and 2B located at the keyhole of AZ31B/DP980 were both situated at the interface layer, where Al from AZ31B may react with Mn to form an Al-Mn phase or with Fe to form a Fe-Al phase. According to the content ratio (Table 7), it was plausible that the interface layer at room temperature could consist of Fe_3_Al, (Fe) solid solution, or LT-Al_11_Mn_4_.

The position of point 3A was located at the keyhole of the AZ31B/316L, positioned right on the periphery of the steel spatter within the weld. The ratios of Mn to Fe and Al to Mg were found to be higher compared to those in the original base metal, indicating an enrichment phenomenon of alloy elements at the Mg/Fe interface, thereby suggesting a potential formation of a new phase. From the quantitative results as presented in Table 8, it can be inferred that both the (Fe) solid solution Fe_3_Al and LT-Al_11_Mn_4_ might coexist in this region.

In summary, Al and Mn elements were found to be enriched at the Mg/Fe interface in the KRA of all three joints. The interfacial compounds consisted of a phase containing Al-Mn particles, as well as Al-Mn and Al-Fe IMCs. Compared to the AZ31B/SK7 joint with a low Mn content, the AZ31B/DP980 joint with a high Mn content exhibited a thicker layer of interfacial compound (Figure 8). However, the contribution of Al-Mn compounds to the mechanical properties of the joints was not found to be significant (Figure 3). The interfacial compound thickness of the AZ31B/316L was smaller compared to the other joints, while the load-bearing capacity of the AZ31B/316L was weaker than that of the other joints (Figure 8). Conversely, Li et al. [32] observed strong metallurgical bonding between Mg/Fe interfaces under TEM examination but found that it generated only a nanoscale interface layer. Therefore, it can be concluded that differences in mechanical properties between these three joints were primarily caused by the difference in the FRA of the weld.

**Figure 9:**
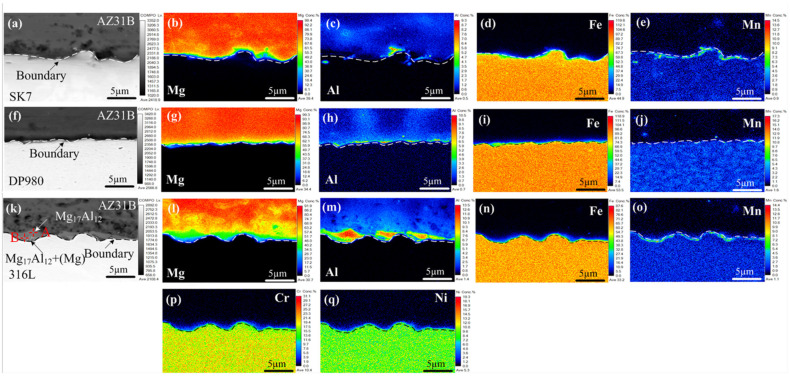
Mapping analysis of the Mg/Fe interface at the FRA: (**a**–**e**) AZ31B/SK7; (**f**–**j**) AZ31B/DP980; (**k**–**q**) AZ31B/316L.

The mapping analysis results at the Mg/Fe interface in front of the three joint welds are presented in Figure 9. Similar to the phenomenon observed at the keyhole interface (Figure 7), enrichment of Al and Mn was also observed at this interface. However, the μ(Al_4_Mn) particle phase was formed but less. Notably, the microstructure of the Mg/Fe interface in front of the AZ31B/316L differed significantly from that of the other two types of joints, as it exhibited a composite structure with multiple layers. Two forms of Al enrichment were observed at the Mg/Fe interface, namely a layered structure and a network structure (Figure 9k). To further ascertain the composition of the interface layer, element composition analysis was conducted at various positions in the FRA in Figure 9k, as shown in Table 9. The main constituents at the interface were found to be Mg and Al, with a significantly higher Al content compared to the AZ31B base metal. Referring to the Mg–Al binary phase diagram [24], it was observed that the interface layer adjacent to the Mg side primarily consisted of Mg_17_Al_12_ IMCs, while the interface layer near the steel side comprised a eutectic mixture of [Mg_17_Al_12_ + α-Mg]. These brittle and thick layers of compounds played a crucial role in contributing to the inferior mechanical properties exhibited by the joint.

**Figure 10:**
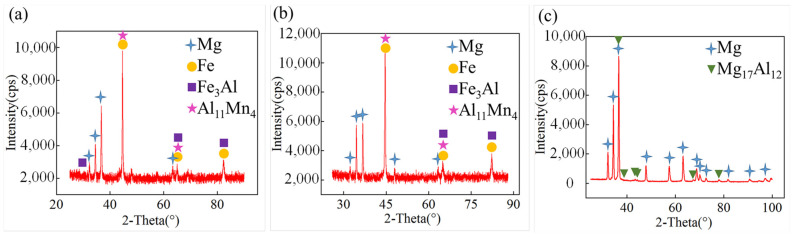
Micro XRD analysis of fractures: (**a**) AZ31B/SK7; (**b**) AZ31B/DP980; (**c**) AZ31B/316L.

To confirm the phase composition of the interface layer, micro XRD analysis was conducted on the fracture surfaces of three joints. The corresponding results are presented in Figure 10. The fracture surfaces of AZ31B/SK7 and AZ31B/DP980 joints not only detected Fe and Mg, but also confirmed the presence of Fe_3_Al and A_11_Mn_4_, indicating that both joint fractures indeed generated the aforementioned Fe–Al and Al–Mn phases at the interface in addition to the residual Mg. Moreover, on the fracture surface of AZ31B/316L joint, significant amounts of Mg_17_Al_12_ compound were detected, indicating that these phases were indeed formed at the FRA during the welding process.

### 3.4. Influence Bonding Mechanism

#### 3.4.1. AZ31B/SK7 and AZ31B/DP980 Joints

**Figure 11:**
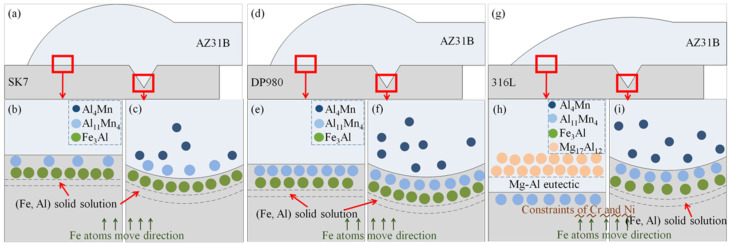
Diagram of interface bonding mechanism: (**a**–**c**) AZ31B/SK7; (**d**–**f**) AZ31B/DP980; (**g**–**i**) AZ31B/316L.

According to the experimental results and analysis, the high-performance joints had similar interface structures, that is, the interface layer at the KRA and FRA of the AZ31B/SK7 and the AZ31B/DP980 joints was a nanometer thick and primarily consisted of Al–Mn compounds, Fe–Al compounds, and Fe-based solid solutions, as shown in Figure 11a–f. All these explained the crucial role of these IMCs formed by high-temperature reactions for joining Mg alloy and steel, as reported in previous studies [33]. The melting of steel under the action of a laser resulted in the accumulation of Mn atoms from the steel and Al atoms from the Mg alloy at the Mg/Fe interface. Upon reaching a temperature of 1538 °C at the interface between the Mg alloy and steel, the Al and molten Fe underwent solidification, resulting in the formation of a (Fe) solid solution. Owing to the lower mixing enthalpy of the Al–Mn phase compared to that of the Fe-Al phase, HT-Al_11_Mn_4_ was initially generated at the leading-edge interface of the weld. Subsequently, HT-Al_11_Mn_4_ decomposed into layered LT-Al_11_Mn_4_ and granular Al_4_Mn at the KRA. As the interface temperature further decreased, Fe_3_Al was formed at the interface [34]. The formation process of the interface at the FRA was similar to that at the KRA, so the interface structure was also similar.

The maximum tensile load of the AZ31B/DP980 joint slightly exceeded that of the AZ31B/SK7 joint due to various factors, including the spreading wettability of Mg on steel and the influence of the interface layer thickness and composition. EPMA suggested that the thickness of the interface layer formed by Mn segregation was only a few nanometers thin. According to the line analysis results shown in Figure 8, it was evident that the interface layer of the AZ31B/DP980 joint exhibited a greater thickness. Previous investigations had demonstrated that an interface layer with a thickness within the range of 10 μm positively influenced the enhancement of joint properties [35]. Additionally, a study had confirmed that the formation of a coherent interface between the Al-Mn compound and the Al-Fe compound contributed significantly towards improving the joint performance [36]. Therefore, the increase in Mn content facilitated the reinforcement of joint bonding. However, the contribution of ultra-thin Al-Mn compounds to the mechanical properties of the joint was found to be insignificant. In conjunction with quantitative analysis results at the interface layer (Table 7), it was observed that Fe-Al compounds, Al-Mn compounds or solid solutions were formed at the Mg/Fe interface of the KRA and FRA within AZ31B/DP980, which played a crucial role in enhancing joint properties.

#### 3.4.2. AZ31B/316L Joint

The interface bonding mechanism of the AZ31B/316L connector is shown in Figure 11g–i. The FRA of the weld conformed to the characteristics of the fusion weld-brazing weld. The steel hardly melted under the action of the small arc current, which led to the limited diffusion of Fe, Mn, Cr, and Ni in the steel to the molten pool. Moreover, Cr and Ni [37,38] are stable components in α-Fe and γ-Fe phases, respectively, so the formation of a Fe-Al phase requires a larger diffusion concentration of Al. However, achieving the necessary conditions for metallurgical reactions of Fe and Al at lower welding temperatures and faster cooling rates was a major challenge. Because Cr and Ni elements existed in the form of solid solutions and stable carbon compounds in steel [39], they tended not to participate in interfacial reactions. Although Al-Cr and Al-Ni compounds had lower mixing enthalpy compared to Al-Mg compounds, they did not form at the interface. Thus, at low temperatures, only a thin layer of Al-Mn compounds was formed at the interface of the FRA. However, Al atoms did not react with enough Fe atoms, and when the temperature was lowered to the eutectic point, the enriched Al atoms and Mg atoms underwent eutectic reactions along the interface, forming a large number of Mg-Al eutectic compounds. With the flow of the molten pool, a thick interfacial layer with a composite structure was formed. Brittle Al-Mg IMCs were formed at the FRA, and the thickness of the interfacial layer reached a micron level, both of which led to a significant reduction in its tensile bearing capacity, as shown in Figure 3. The wetting angle of AZ31B on 316L was significantly smaller than that of SK7, which can be attributed to the presence of Cr [18] and Ni [21] elements. These elements helped to form a smooth passivation film on the steel surface, thereby improving the diffusion wettability of the Mg alloy on the steel. Nevertheless, the performance of AZ31B/316L was the worst of the three joints; the observation highlighted the critical role that the phase composition of the interfacial compounds played in determining the performance of the joint.

At the KRA of AZ31B/316L, the action of the laser beam partially melted the steel, which was in line with the characteristics of fusion welding (Figure 7k). When the temperature at the keyhole exceeds the boiling point of Mg (1090 °C), the Mg will be violently vaporized, and the Al at the high boiling point (2327 °C) will be forced to segregate and accumulate. Due to the action of a high-energy laser, the energy obtained by Fe and Mn atoms far exceeded their own diffusion activation energy, and their diffusion rate increased rapidly, thus weakening the stabilizing effect of Cr and Ni on α-Fe and γ-Fe. Therefore, a metallurgical reaction will be carried out with the segregated aggregated Al at high temperatures to form nanoscale Al_4_Mn particles and Al_11_Mn_4_ and Fe_3_Al IMCs. The composition of the interface layer at the KRA was similar to that of the other two joints. In conclusion, the formation of a thick and brittle eutectic structure at the FRA of AZ31B/316L was the key to the deterioration in the tensile properties of the joint; therefore, the influence of the Al element should be avoided as far as possible under the low temperature welding conditions of AZ31B and 316L.

## 4. Conclusions

In this paper, the high-performance laser-GTAW welding of Mg and steel was realized by adjusting the heat source offset and the laser-arc malposition. The direct welding lap joints of AZ31B and three steels (SK7, DP980, 316L) under the same welding parameters were obtained by this method. By comparing the three joints, the following conclusions can be drawn:

(1) The welds of the three kinds of joints were well formed. The spreading wettability of magnesium on 316L was the best; that is, the weld width was 6.6mm, the weld depth was 1.9mm, and the wetting angle was 51°.
(2) The tensile loads for the AZ31B/SK7, AZ31B/DP980, and AZ31B/316L joints were measured as 283 N/mm, 285 N/mm, and 115 N/mm, respectively.
(3) For Mg/steel direct welded joints, the formation of a nanoscale interface layer mainly composed of the Al-Mn phase and Fe-Al phase in the FRA and KRA made the joints exhibit better performance, and the increase in Mn content was conducive to the formation of a thicker and more continuous interface layer.
(4) The binding of Cr and Ni on Fe atoms in the FRA at a lower reaction temperature instead promoted the formation of a large number of brittle Mg-Al eutectic and micron-scale interface layers, resulting in a sharp deterioration in the performance of AZ31B/316L joints.

## Figures and Tables

**Table 1:** Main chemical composition of substrates (wt.%).

	C	Si	Mn	Zn	Al	Cr	Ni	Mg	Fe
AZ31B	-	-	0.32	0.61	3.05	-	-	Bal.	-
SK7	0.66	0.25	0.47	-	-	-	-	-	Bal.
DP980	0.19	0.31	2.66	-	-	-	-	-	Bal.
316L	0.03	0.42	2.0	-	-	16.0~18.0	12.0~16.0	-	Bal.

**Table 2:** Welding parameters.

Parameter	Parameter Value
Average pulse power (P_L_)	300 W
GTAW current (I)	80 A
Distance between tungsten tip and magnesium alloy edge (D_o_)	3 mm
Height of tungsten tip from Mg alloy upper surface (H_T_)	1.5 mm
Horizontal distance between tungsten tip and laser beam in O-X direction (D_la_)	1.5 mm
Horizontal distance between tungsten tip and laser beam in O-Y direction (Malp)	1 mm
Welding speed	600 mm/min
Protective gas flow (Ar, purity: 99.99%)	15 L/min
Pulse frequency	30 Hz
Pulse width	2.6 ms
Current mode of GTAW	Alternating current

**Table 3:** Quantitative point analysis results (at. %) of positions marked in Figure 5a–c.

Points	Fe	Mn	Mg	Al	Possible Phase
1A	65.05	1.18	28.39	4.39	Fe_3_Al
1B	0.06	0.31	96.61	2.36	α-Mg
1C	0.14	0.09	96.56	2.92	α-Mg
1D	69.82	0.90	29.09	0.02	(Fe), α-Mg
1E	1.09	0.10	95.45	3.02	α-Mg
1F	92.21	0.70	6.27	0.29	(Fe)
1G	0.14	0.28	96.56	2.92	α-Mg

**Table 4:** Quantitative point analysis results (at. %) of positions marked in Figure 5d–f.

Points	Fe	Mn	Mg	Al	Possible Phase
2A	0.10	5.77	82.64	11.01	Al_11_(Mn,Fe)_4_
2B	0.30	0.04	96.45	2.28	α-Mg
2C	0.93	0.04	89.23	9.29	Mg_17_Al_12_
2D	0.78	0.22	88.72	8.76	Mg_17_Al_12_
2E	32.50	1.17	59.41	5.85	Fe_3_Al
2F	0.39	0.18	95.75	3.38	α-Mg

**Table 5:** Quantitative point analysis results (at. %) of positions marked in Figure 5g–i.

Points	Fe	Mn	Cr	Ni	Mg	Al	Possible Phase
3A	0.06	0.06	0.05	0.01	83.18	12.82	Mg_17_Al_12_
3B	4.69	0.63	1.26	1.01	67.14	21.78	Mg_17_Al_12_
3C	0.09	0.01	0.02	0.02	96.13	2.71	α-Mg
3D	0.04	0.07	0.01	0	95.09	4.10	α-Mg
3E	7.37	1.88	1.62	1.21	76.85	11.00	Mg_17_Al_12_
3F	32.00	3.91	8.40	4.35	23.75	26.20	Al_11_(Mn,Fe)_4_, Fe_3_Al
3G	52.47	3.60	13.99	7.23	6.92	15.09	Al_11_(Mn,Fe)_4_, Fe_3_Al
3H	52.77	3.29	13.95	7.14	6.02	15.83	Al_11_(Mn,Fe)_4_, Fe_3_Al
3I	49.78	2.45	11.96	5.87	13.18	15.49	Mg_17_Al_12_, Fe_3_Al
3J	3.21	0.62	1.15	0.96	81.55	11.49	Mg_17_Al_12_

**Table 6:** Results of quantitative point analysis (at. %) at the KRA of AZ31B/SK7 (Figure 8a).

Points	Fe	Mn	Al	Mg	Possible Phase
1A	66.59	1.38	15.33	16.71	α-Fe, LT-Al_11_(Mn, Fe)_4_
1B	98.45	0.95	0.07	0.95	(Fe)
1C	68.61	1.26	14.65	15.48	α-Fe, LT-Al_11_(Mn, Fe)_4_
1D	11.85	0.35	12.99	74.81	α-Mg, Fe_3_Al

**Table 7:** Results of quantitative point analysis (at. %) at the KRA of AZ31B/DP980 (Figure 8b).

Points	Fe	Mn	Al	Mg	Possible Phase
2A	57.39	1.79	6.03	34.79	α-Fe, Fe_3_Al, LT-Al_11_(Mn, Fe)_4_
2B	46.10	1.58	5.85	46.46	α-Fe, Fe_3_Al, LT-Al_11_(Mn, Fe)_4_
2C	12.54	0.50	2.39	84.57	α-Mg
2D	96.45	2.36	0.15	1.03	(Fe)

**Table 8:** Results of quantitative point analysis (at. %) at the KRA of AZ31B/316L (Figure 8c).

Points	Fe	Mn	Cr	Ni	Mg	Al	Possible Phase
3A	13.94	0.93	3.30	1.46	75.09	5.28	Fe_3_Al, LT-Al_11_(Mn, Fe)_4_
3B	0.76	0.12	0.34	0.07	98.49	0.22	α-Mg
3C	57.20	1.25	14.55	7.63	18.78	0.59	(Fe)
3D	0.19	0.05	0.09	0.01	97.39	2.26	α-Mg

**Table 9:** Results of quantitative point analysis (at. %) at the FRA of AZ31B/316L (Figure 9k).

Points	Fe	Mn	Cr	Ni	Mg	Al	Possible Phase
A	0.82	0.21	0.41	0.15	70.39	28.02	Mg_17_Al_12_
B	11.15	2.12	3.51	1.32	65.49	16.18	Eutectic structure [Mg_17_Al_12_ + α-Mg]

## Data Availability

Data are contained within the article.
